# From 2D cultures to 3D systems: evolving cancer models at the interface of functional precision medicine and theranostics

**DOI:** 10.7150/thno.127053

**Published:** 2026-01-21

**Authors:** Yizheng Zhang, Naray Payab, Bettina Weigelin, Christian M. Schürch

**Affiliations:** 1 Department of Pathology and Neuropathology, University Hospital and Comprehensive Cancer Center Tübingen, Tübingen, Germany.; 2 Werner Siemens Imaging Center, Department of Preclinical Imaging and Radiopharmacy, University of Tübingen, Germany.; 3 Cluster of Excellence iFIT (EXC 2180) "Image-guided and Functionally Instructed Tumor Therapies", University of Tübingen, Germany.

**Keywords:** functional precision medicine, cell culture, spheroids, organoids, cancer-on-a-chip, tumor explants, PDX, tumor microenvironment

## Abstract

Advances in patient-derived cancer models are pushing precision oncology by linking functional testing directly to therapeutic decision-making. Traditional two-dimensional (2D) cancer cell culture systems have long served as accessible tools for studying cancer biology and drug responses, but their inability to replicate the complexity of the tumor microenvironment limits their translational value. In recent years, advances in culture and imaging technologies have enabled the development of three-dimensional (3D) cancer models, such as spheroids, organoids, and patient-derived explants, that more accurately represent tumor architecture and behavior *in vivo*. These models better capture cell-cell and cell-ECM interactions and allow to study immune-tumor dynamics, providing critical insights into therapeutic efficacy and drug resistance of chemotherapies, targeted therapies, and immunotherapies. Notably, the integration of 3D modeling with functional precision medicine approaches, such as *ex vivo* drug screening using patient-derived samples, has opened new avenues for individualized cancer treatment. Coupling these advanced models with advanced imaging readouts for spatially resolved and functional analysis further transforms them into quantitative theranostic platforms that link biological mechanisms to clinical decision-making. In this review, we explore the evolution from 2D to 3D cancer models, examine their respective advantages and limitations, and highlight their role in advancing functional precision oncology and immuno-theranostics.

## Introduction

Cancer is a long-lasting global health challenge and the gap between preclinical promise and clinical benefit remains large 1,2. One reason for this is the fact that most of the commonly used models do not replicate the cellular heterogeneity, spatial organization, and dynamic signal transduction of the human tumor microenvironment (TME), conflating treatment response. In addition to this, the TME is significantly influenced by mechanobiological factors, including extracellular matrix (ECM) stiffness, physical confinement, and shear stress from fluid flow. These mechanical drivers are responsible for the migration of cancer cells; immune-cell trafficking; delivery of foreign products, including drugs; and for the development of treatment resistant TMEs 3-6. These considerations are particularly relevant when selecting experimental models since 2D cultures, 3D matrices, organoids, tissue slices, and microfluidic systems each vary significantly in their ability to recapitulate physiologically relevant mechanical contexts. For that reason, treatments that appear appealing *in vitro* or in animal models are often poorly reported in patients. This inspires a renewed emphasis on models that retain better the native human tumor biology 7,8.

Over the last ten years, 3D culture systems, such as spheroids, organoids, scaffold-based constructs, microfluidic cancer-on-chip platforms and patient-derived xenografts (PDX), have expanded our toolbox. Each model represents a balancing act that involves scalability, biological realism, and translational applicability. However, none by itself can fully recapitulate the intact human TME with its stromal, vascular and immune constituents arranged in native architecture. Importantly, this limitation is particularly consequential for immuno-oncology, since the spatial context and quality of immune-tumor interactions are pivotal.

Functional Precision Medicine (FPM) is a developing aspect of personalized oncology seeking to match patients with a more potent regimen of drugs through testing the drug on their own tumor cells 9. In contrast to previous precision medicine approaches based on genomic profiling of patients and predicting drug sensitivity, FPM assesses the real-time functional behavior of living cancer cells when exposed to therapeutic agents 9,10. This enables direct assessment of drug effectiveness in clinically relevant patient scenarios, particularly when genomic alterations are absent or non-actionable.

Among the crucial factors making FPM successful is the development of biologically relevant culture systems or cancer models for cancer. HeLa cells were isolated as the first immortalized human cancer cell line in 1951 and transformed the field of *in vitro* cancer studies 11. Since then, 2D cell cultures have evolved as cost-effective and important instruments of exploration of basic cancer biology, drug discovery, and molecular pathway detection with the help of model constructs 12. These models have been used in the investigation of cancer cell behavior and to research potential therapies in lab-controlled microenvironments. Nonetheless, their intrinsic simplicity has considerable drawbacks, since simplistic models struggle to mimic the 3D geometry and complex TME including interactions of the cell-cell and cell-ECM 12,13. More complex cancer models such as tumor spheroids, organoids, cancer-on-a-chip systems, and bioreactors better mimic from duplicating cancer complexity in the TME as well as cellular interactions within the TME. With 3D culture systems that can also adopt patient-specific features of the TME, such as patient-derived organoids (PDOs) and patient-derived tissue slice cultures, these technologies have become valuable assets for personalized medicine 13. Moreover, PDX models are commonly used to serve as *in vivo* 3D models for drug screening and mechanistic studies 14. However, PDX models are also limited in their clinical applicability because engraftment and expansion require weeks to months, which is too slow for many treatment-decision timelines 15. The species-specific immune cellular components and metabolic differences could also reduce the accuracy of prediction 16.

This review discusses the current state of science of cancer models within FPM context, including their increasing significance within immuno-oncology and theranostic applications. We describe conventional 2D and 3D culture systems in order to compare strengths and limitations, and discuss recent developments in patient-derived tissue explants and dynamic culture platforms that can directly assess therapeutic and immune responses within a preserved TME. Additionally, we demonstrate to what extent spatially resolved and dynamic analytical modalities, such as live microscopy and multiplex tissue imaging, can complement these models, connecting molecular mechanisms with therapeutic efficacy *in situ*. No model is ever perfect, but there are systems now in practice which can facilitate the selection of the most appropriate approach for the biological question or clinical decision. Together, these developments emphasize the extent to which advanced cancer models are changing the interface between experimental studies and clinical decision-making to a theranostic field, one that integrates functional testing, mechanistic knowledge and individualized therapeutic planning within one experimental framework.

## Functional Precision Medicine

Accurately predicting cancer behavior requires not only static genomic, proteomic, and metabolic profiles but also an understanding of how these components interact across different cellular states 17. While genomic profiling reveals accumulated mutations, it does not capture dynamic functional responses 18. FPM, which tests live patient-derived materials, provides more direct and actionable insight into therapeutic sensitivities and has shown clinical value, particularly for patients without actionable mutations or those resistant to standard therapies 19. As immuno-oncology advances, models that incorporate interactions between tumor and immune cells enable the evaluation of checkpoint inhibitors, adoptive cell therapies, and vaccines under near-physiologic conditions, expanding FPM from drug-response testing to predictive immune theranostics.

The idea of FPM originated decades ago with assays that exposed patient-derived samples to drugs and measured cell death, initially focusing mainly on hematologic cancers 20-22. Advances in culture systems have since expanded FPM from simple drug-sensitivity testing to evaluating TME influences, immune interactions, and therapy-induced cellular changes 23,24. Modern platforms integrate immune-competent co-cultures, tumor explants, and functional assays such as dynamic BH3 protein profiling and lactate dehydrogenase (LDH) release assays to characterize drug-induced apoptotic priming and treatment-associated cytotoxic responses 25-27. Integration of multimodal data, supported by artificial intelligence (AI)-driven analysis, now enables biomarker discovery and therapeutic target identification that can be validated in patient-derived models, positioning FPM firmly within the theranostic framework 28,29. In parallel, advances in machine learning enable automated analysis of high-content imaging, single-cell phenotyping, and complex drug response patterns. Integrative models that combine multiomics, clinical, and biological data enhance patient stratification and improve predictions of disease progression, treatment response, and risk, thereby increasing the clinical utility of FPM platforms 30,31.

**Figure 1:**
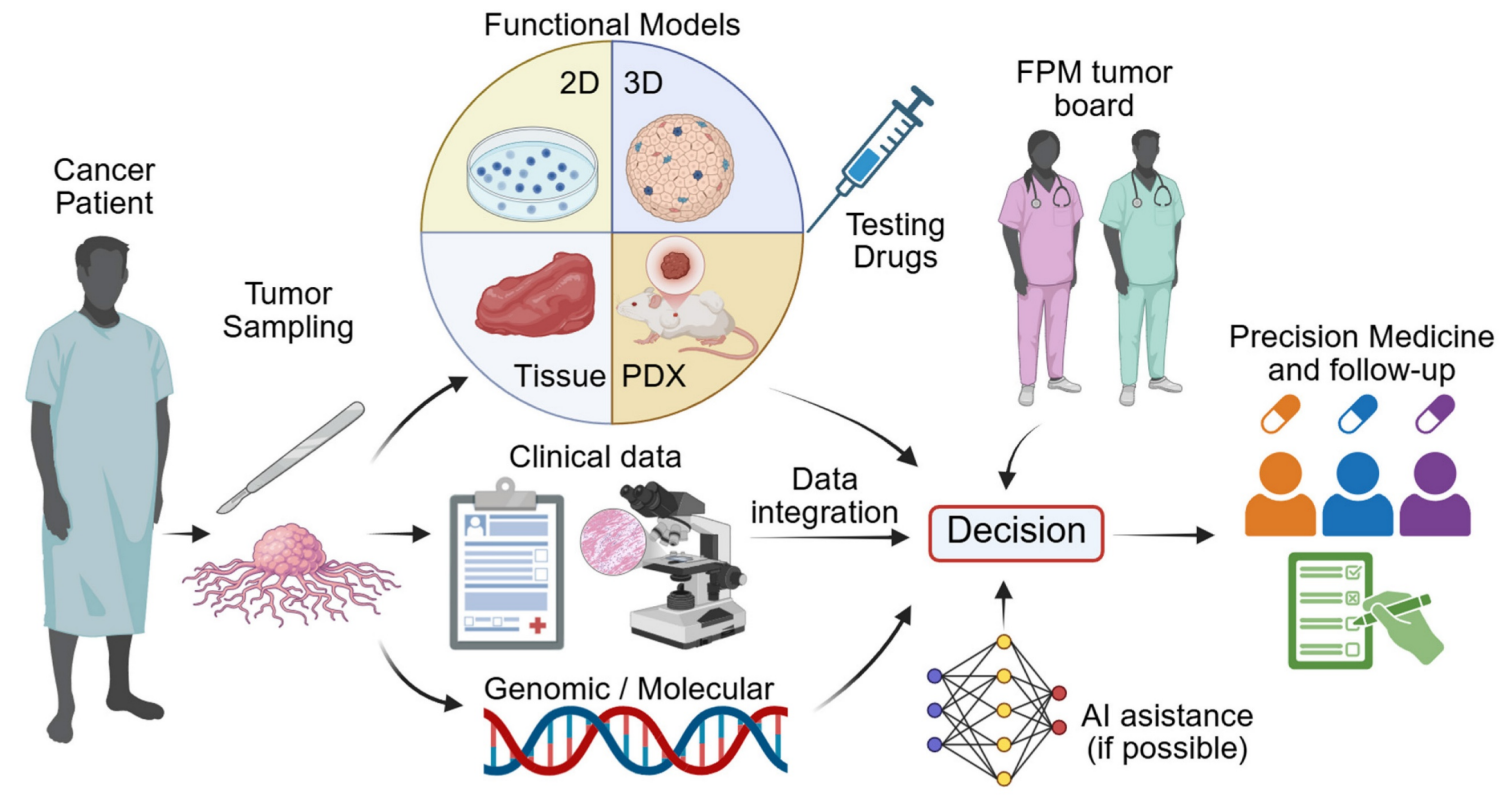
** FPM in Cancer Management.** Generally, advanced-stage cancer patients enroll at treatment centers, and tumor tissue is collected via surgery or biopsy. Following that, the functional models are then selected according to tumor type and urgency of treatment. The FPM tumor board reviews drug sensitivity results along clinical and genomic data with AI assistance to guide precision therapy and follow-up.

A standard FPM workflow consists of patient enrollment, tumor sampling and generation of patient-derived 2D or 3D models for drug sensitivity testing, with results being integrated alongside clinical and molecular data and reviewed by an FPM tumor board. More recent pipelines leverage sophisticated 3D and tissue-explant systems to better preserve the TME and immune context. Microfluidic or perfused bioreactors also contribute to maintenance of immune-tumor interactions and support longitudinal functional readouts. Ultimately the effectiveness of FPM is assessed through iterative comparison of model predictions with patient outcomes (**Figure 1**).

As functional assays diversify, the choice of model system becomes central to both technical feasibility and diagnostic relevance. In a theranostic context, the model itself serves as a biosensor, translating biological complexity into measurable treatment response. The following sections therefore compare available model formats and illustrate how their design, ranging from simple 2D cultures to dynamic explant bioreactors, determines their suitability for personalized therapy and immuno-oncology applications.

## Patient-Derived Cancer Models for Drug Sensitivity Evaluation

**Figure 2:**
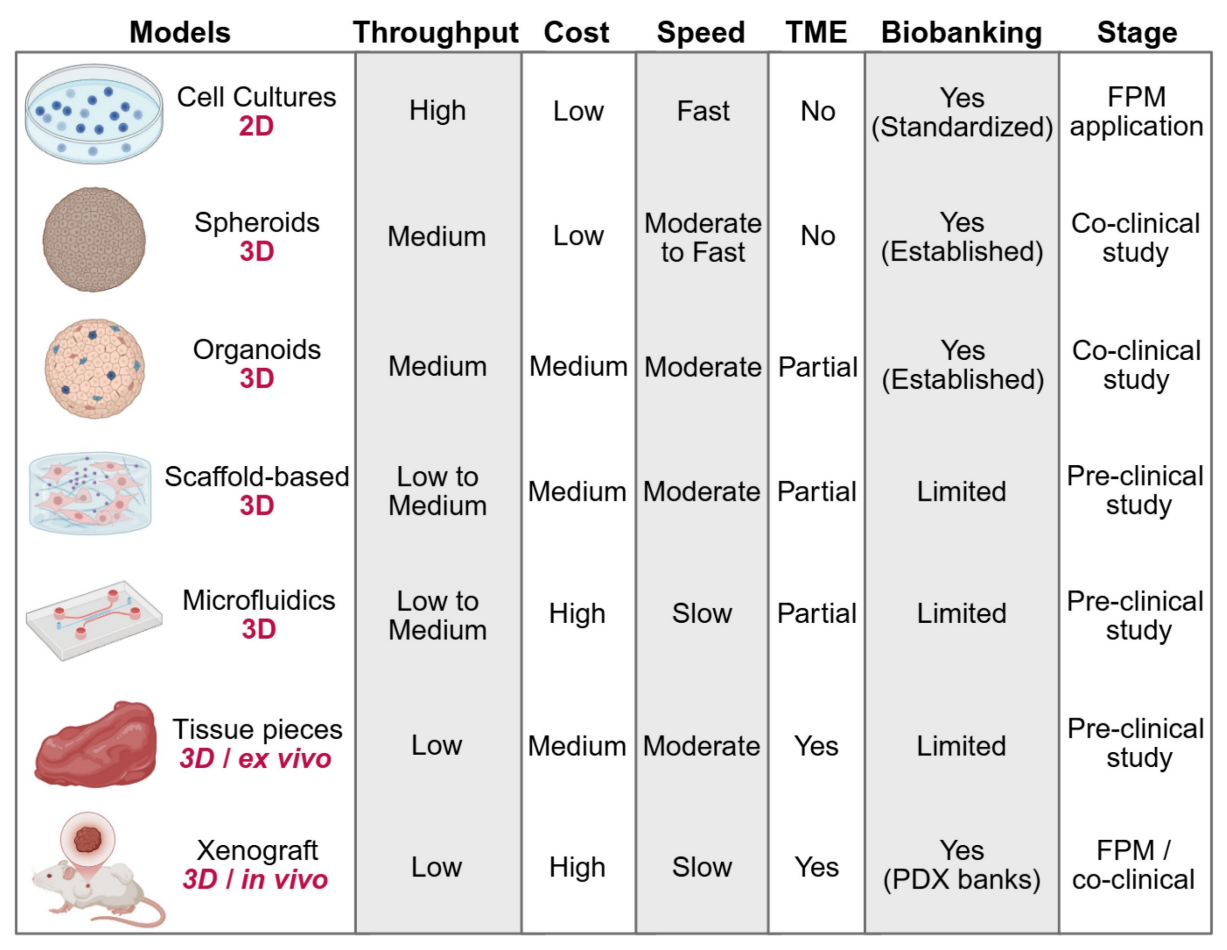
** Comparison of Cancer Models for FPM.** Models are compared by dimensionality, throughput, cost, culture speed, TME preservation, biobanking potential, and stage of clinical application. The FPM application stage indicates models used to directly inform patient treatment decisions. The co-clinical study stage refers to models used alongside standard patient treatment to evaluate therapeutic responses. The preclinical study stage includes models used for basic and translational research. Additional study details are provided in** Table 1**.

The evolution of various cancer models has helped FPM by allowing direct measures of patient-specific drug responses. Its cost, throughput, speed, and preservation of TME characteristics vary for each model, influencing its application in different clinical settings. In immuno-oncology, these properties also determine whether a model is capable of sustaining meaningful interactions between tumor, stromal and immune compartments, which is a prerequisite for evaluating checkpoint blockade, adoptive cell transfer or cytokine-modulating therapies. 2D cultures are still fast, cost-effective, but have operationally straightforward nature, thus aiding in timely clinical decisions in FPM pipelines. Nevertheless, due to their low structural complexity and lack of immune context their applicability is restricted to either cytotoxic or targeted-agent screening purposes. By contrast, 3D models better mimic both tumor architecture and heterogeneity and yield accurate therapy predictions. By combining various cell types together and sustaining spatial gradients, 3D models also support immune infiltration and drug distribution, which are increasingly important parameters of immuno-oncology testing and theranostic assay development. While their costs, complexity, and longer duration can restrict their applications in the clinic and they are valuable tools for mechanistic finding, biomarker discovery and selective FPM applications 19. We compare these models and describe their advantages and disadvantages, as illustrated in **Figure 2**, **Table 1** and also given in the following text, and how they help to influence FPM. Particular emphasis is added to new patient-derived 3D systems, such as organoids, explants, and perfused cultures, which link mechanistic research and clinical decision-making and represent the way in which cancer modeling is advancing into a theranostic field.

### 2D cell culture

2D monolayer cultures remain the simplest and most widely used patient-derived cancer models. They are inexpensive and suitable for large-scale drug screening but inherently lack the 3D architecture, stromal signaling, and immune context that shape real tumor responses 28. This loss of microenvironmental signaling can limit their predictive accuracy for complex tumor behaviors, including metabolic dependencies and interactions with stromal or immune cells, and may underestimate drug resistance mechanisms that depend on tissue architecture 32. Moreover, inconsistent isolation procedures and low culture initiation success rates hamper the reproducibility of 2D cultures 33.

Suspension-based 2D models were initially developed for hematological malignancies, where tumor cells naturally exist as single-cell suspensions compatible with high-throughput drug screening 29,34. Several clinical studies have demonstrated that such functional testing can guide therapy selection and improve outcomes in relapsed or refractory leukemia and lymphoma patients 22,35. In acute myeloid leukemia (AML), which included 78 relapsed and 41 refractory cases, 2D suspension cultures were used for screening 515 drugs, which promoted clinical response in 59% of patients that received treatment including 45% complete remission 36 (**Table 1**). Suspension-based 2D models in hematologic cancers are gaining popularity in the clinic, and there is increasing evidence that they can be used in FPM on a regular basis 22,36,37.

Likewise, monolayer 2D cultures are among the most technically accessible models for studying solid tumors in FPM. Several clinical studies have demonstrated their utility in predicting therapeutic responses 10 (**Table 1**). As example, in one large-scale study involving 568 tumor biopsies from various solid cancer types, drug sensitivity was quantified using 2D monolayer cultures with 6 tyrosine kinase inhibitors, and the observed sensitivities were found to reflect actual clinical responses 38. Thus, despite their simplicity, 2D monolayer models remain relevant and effective tools for rapid, cost-effective screening in FPM, although their lack of microenvironmental context remains an important limitation to consider when interpreting results.

### 3D spheroids

3D spheroids are widely recognized as one of the earliest and most fundamental forms of 3D cell culture. Cells in spheroids aggregate and interact in all three dimensions. Although most spheroid models lack the cellular heterogeneity and architectural complexity of native tumor tissues, they were developed to overcome the limitations of traditional 2D cultures. Spheroids better mimic critical tumor characteristics, including cell-cell interactions, oxygen gradients, drug penetration, and resistance patterns 39,40. Such properties are of paramount importance in the field of FPM for more accurately predicting therapeutic responses. When co-cultured with autologous immune or stromal cells, spheroids can also recapitulate immune cell infiltration and cytokine signaling, providing a simple but useful assay for immunotherapy evaluation.

Beyond conventional low-adhesion and hanging-drop techniques, emerging material-engineering approaches based on liquid-liquid phase separation (LLPS) offer new strategies for controlling spheroid formation. LLPS, a process in which a homogeneous mixture separates into distinct liquid phases, has been recognized in biological systems as a mechanism for organizing non-membrane cellular compartments and coordinating biochemical processes. When applied to soft material engineering, LLPS-based systems enable customizable microenvironments that support controlled cellular organization and may facilitate the generation of more physiologically relevant spheroid structures for cancer modeling 41.

Spheroids, as the simplest, most cost-effective, and rapid-to-establish 3D cancer models, are widely used in clinical FPM studies. Their ability to better mimic the tumor architecture compared to 2D cultures makes them valuable for drug sensitivity testing. In a lung cancer study, cancer spheroids generated from 20 patient biopsies were used to evaluate responses to 6 tyrosine kinase inhibitors, achieving a strong predictive accuracy of 85% in co-clinical trials 42 (**Table 1**). In another study involving 44 newly diagnosed ovarian cancer patients, drug testing with six chemotherapy agents based on 3D spheroids reached an overall prediction accuracy of 89% 43. Collectively, these studies position spheroids as a practical bridge between high-throughput drug screening and more advanced patient-derived 3D or *ex vivo* systems, although the absence of an TME constrains their relevance for immunotherapy evaluation.

### Organoids

Organoids have greater structural and biological complexity compared to spheroids, preserving the histological, genetic, and phenotypic features of the original tumor and their ability to self-organize into tissue-like structures. Long-term expansion is maintained and they can incorporate multiple cell types, which better resemble the heterogeneous TME 44. This complexity allows the examination of immunotherapies that depend on the interaction of immune and cancer cells 45. Such characteristics further promote the accuracy of clinical drug response prediction, improve the modeling of tumor heterogeneity, and offer a basis for personalized treatment strategies with higher translational relevance.

Recent work has also contributed to the development of PDOs as a central instrument in FPM. Novel investigations suggest a transition from genomics-only guidance toward *ex vivo* functional testing on living patient-derived tumor models, including PDOs, directly exposed to therapeutics to characterize patient-driven responses 46. Meanwhile, recent developments in biofabrication and organoid-microfluidic system integration have yielded a number of cancer-on-a-chip platforms capable of more closely mimicking the complexity of TME and to conduct high-content assessment of therapeutic effectiveness 47. Together, these advances have highlighted a growing place for PDO-based systems in next-generation cancer modeling and personalized therapy design.

PDOs have also recently emerged as powerful co-clinical models for the analysis of functional drugs for solid tumors because they recapitulate important features of patient tumors. In a study on metastatic gastrointestinal cancers, 29 biopsy samples from 21 patients were used to generate organoids and screen 55 drugs, achieving a high predictive accuracy with 100% sensitivity and 93% specificity 48. Similarly, in breast cancer, PDOs derived from 35 patients were tested on a panel of 49 drugs that included immunotherapies, achieving 82.35% sensitivity and 69.23% specificity to predict clinical response 49 (**Table 1**). Currently, due to their broad application and extensive study, PDOs show strong potential to become the most widely used 3D model for FPM. Their compatibility with imaging, omics, and multiplex profiling further positions PDOs as a central model of emerging theranostic workflows.

### Scaffold-based cancer models

3D culture systems based on scaffolds fall under the category of 3D models that include physical frameworks to help and organize cell growth. Scaffold-based systems deliver superior mechanical support, a clear architecture, and much higher reproducibility 50. Several scaffold types have been established, all of which have their advantages: synthetic polymer scaffolds are easily adaptable and are suitable for large-scale manufacture, but surface coatings are necessary for better biocompatibility, hydrogels imitate ECM characteristics and facilitate cell viability, and decellularized scaffolds retain native tissue architecture and allow cell function 51. Few significant limitations also appear. Natural polymer scaffolds have significant batch-to-batch variability as well as immune response induction. By comparison, synthetic degradable polymers result in more uniform consistency, but have potential to impair biocompatibility as cytotoxic degradation products can arise and also have limited support for their native cell functions. Hydrogels, while tunable, often have low mechanical strength and long-term stability. Decellularized tissues also experience issues with availability, substantial batch-to-batch variation, and residual xenogenic components which compromise the reproducibility and translational reliability 51. These models nevertheless represent a unique recapitulation of the structural TME and are useful for studies where biomechanical characteristics of the tissue are of benefit. Scaffold systems can further model gradients of cytokines or cell distributions that are also relevant in studies on immune exclusion and mechanical mechanisms of immune cell trafficking in tissues, when seeded with immune or endothelial cells 52,53.

Currently, scaffold-based cancer models are primarily investigated in the fields of materials development and preclinical research. These systems show considerable promise in evaluating patient-specific drug responses across various tumor types. For instance, polymer scaffold based models for breast cancer and hydrogel based models for prostate cancer have demonstrated the feasibility of drug screening in a personalized context 54,55. In colorectal cancer , decellularized tissue scaffold-based cancer models constructed from 23 patient samples were used to assess responses to 5-fluorouracil (5-FU) or FOLFIRI (folinic acid, fluorouracil, and irinotecan), providing a patient-specific platform for functional drug evaluation 56 (**Table 1**). Collectively, these studies highlight the potential of scaffold-based systems in advancing functional precision oncology. By combining mechanical tunability with cellular complexity, scaffold platforms may serve as modular components of hybrid 3D *ex vivo* systems, bridging material science and (immuno-) oncology within a theranostic framework.

### Cancer-on-a-chip

Cancer-on-a-Chip (CoC) is an *in vitro* model based on microfluidics which is developed to more accurately mimic the TME. The challenges of such systems include a lack of standardization, high cost, and low throughput, but also bring advantages in a different way. Their underlying principle is based on fluid flow to create shear stress, oxygen gradients, and controlled nutrient delivery in a way that imitates the physical, chemical, and biological environment of cancer tissues in a controlled, small-scale fashion 57,58. The combination of the patient-derived cells with the ECM provides the means for monitoring the performance of real-time cell behavior, how cells interact and are activated or inhibited by drugs to identify and control behavior and drug response. Some of those more sophisticated models have the capability to incorporate multiple organ-like compartments on a single chip, thus making these models valuable for basic studies and FPM application in specific applications 57. For immuno-oncology, CoC systems allow spatial separation of immune and tumor compartments connected by microchannels, permitting quantitative analysis of immune-cell migration, tumor infiltration, and cytokine gradients under defined flow conditions 59, which is an emerging frontier for functional immunotherapy testing.

Nonetheless, while still in its infancy, CoC technology is proceeding fast, underscoring its potential for personalized immunotherapy in increasing numbers of new studies. For instance, in a preclinical breast cancer study conducted from 2 breast cancer patients' samples, a CoC was used to measure CAR-T cell therapy, facilitating a target dose-response assessment on an individual basis for both efficacy and safety 60. Similarly, a trial of 12 lung cancer patients utilizing CoC to model anti-PD-1 immunotherapy enabled us to evaluate patient-specific drug response 61 (**Table 1**). These proof of concept studies demonstrate how CoC technologies can be developed into miniaturized theranostic devices for the application of real-time, immune-competent drug testing.

Although microfluidic platforms can improve microenvironmental regulation at the level of microenvironment, they possess small tissue masses. To preserve native tumor architecture and immune cell assembly on a greater scale, *ex vivo* tumor explants and perfusion-culture bioreactor technologies, which maintain spatio-functional integrity of patient tissue and facilitate dynamic therapy evaluation have been investigated.

### Tumor explants

Tumor explant cultures, consisting of small tissue fragments and slices, narrow the divide between *in vitro* and *in vivo* models by preserving the native tumor architecture and cellular composition of the TME. By preserving innate cell-cell and cell-ECM interactions, such cultures maintain essential spatial relationships among cancer, stromal, and immune cells that are otherwise lost during tissue dissociation 62. Tumor tissues are normally cut into small fragments or thin slices to improve viability and oxygenation, which act as a pathway to nutrient diffusion and waste removal. Nevertheless, in conventional static cultures that are only temporarily viable, the gradients of oxygen and nutrients result in progressive cell death and loss of immune function.

To address these restrictions, perfusion-based culture systems are fabricated for continuous circulation of medium throughout or across the tissue. These bioreactor platforms prolong culture duration from days to weeks while preserving tissue morphology, metabolic activity, and immune competence 63,64. The system has varied from basic microfluidic flow-through platforms to perfusion bioreactors, which trade throughput with the need for physiological fidelity in the system. Perfused explants have also been extensively applied for evaluation of chemotherapy, targeted therapy and immunotherapy responses for various tumor types including glioblastoma, pancreatic cancer and lymphoma 62,65,66.

In immuno-oncology, perfused tumor and lymphoid explants offer a unique opportunity to evaluate checkpoint inhibitors, CAR T cells, or oncolytic viruses in the patient's TME. Studies in glioblastoma and melanoma have revealed that both *ex vivo* immune activation and T cell infiltration patterns observed in perfused explants correlate with clinical response 62,67. Imaging T cell migration and tumor cell killing in real time, within such systems, adds a mechanistic layer linking immune dynamics to therapeutic outcome.

Consequently, from a theranostic point of view, the perfused tissue models have emerged as living diagnostic devices that generate clinically relevant functional information on therapy efficacy and provide molecular data for biomarker discovery. Their capacity to extract patient-specific readouts within actionable timelines renders them an exciting tool for the FPM pipeline, complementing organoids and xenografts rather than displacing them.

### Xenografts

Whereas perfused tumor explants allow for the viability of human tissue beyond the body, PDX represent their *in vivo* counterpart where patient tissue is engrafted into immunodeficient mice to study tumor growth and therapy response under systemic physiological conditions 68,69. PDX models are a unique *in vivo* platform to explore drug distribution, metabolism, elimination, and toxicity. By accurately simulating the native tumor biology and treatment response, these models provide valuable insights 14,19.

Humanized PDX systems allow the reconstitution of human immune components in the host mouse and, with that, partial modeling of tumor-immune interactions. These features render them especially important for preclinical immunotherapy studies that can be used for the screening of immune checkpoint inhibitors, CAR-T cell therapies, or combination regimens in a human-like immune context 70.

However, the lengthy engraftment process, high cost, variable success rates, and ethical concerns limit the feasibility of PDX models for routine clinical application in FPM 71. Nevertheless, PDX models are an important way to analyze drug sensitivity and to investigate the mechanism of drug resistance, providing a viable *in vivo* platform for validation of new therapeutics and drug delivery strategies. In gallbladder cancer, a clinical study involving 12 patients demonstrated that PDX-guided chemotherapy significantly improved overall survival and disease-free survival 72. Similarly, in head and neck squamous cell carcinoma, a cohort of 49 PDX models was used to evaluate individual responses to cetuximab 73 (**Table 1**). To enhance their translational application in FPM, future efforts should focus on optimizing and standardizing PDX workflows to better meet clinical demands.

## Spatial and functional readouts for theranostic applications

With increasingly sophisticated model systems available, equal attention must be given to the technologies that are used to extract meaningful data from these models. As the complexity of 3D and *ex vivo* culture strategies increases, advancements in technologies to quantify spatial and functional parameters of (immune) cell response in tumor tissues have been made 74,75. High-content microscopy, multiplex immunofluorescence, and spatial transcriptomics now permit quantitative mapping of drug responses within intact tissues 76-78. Such approaches show spatial heterogeneity, for example differential drug penetration, localized apoptosis, or immune exclusion, that bulk assays cannot capture. In combination with confocal or multiphoton time-lapse imaging, they allow visualization of dynamic phenomena, such as immune-cell trafficking and tumor-cell killing in real time.

**Figure 3:**
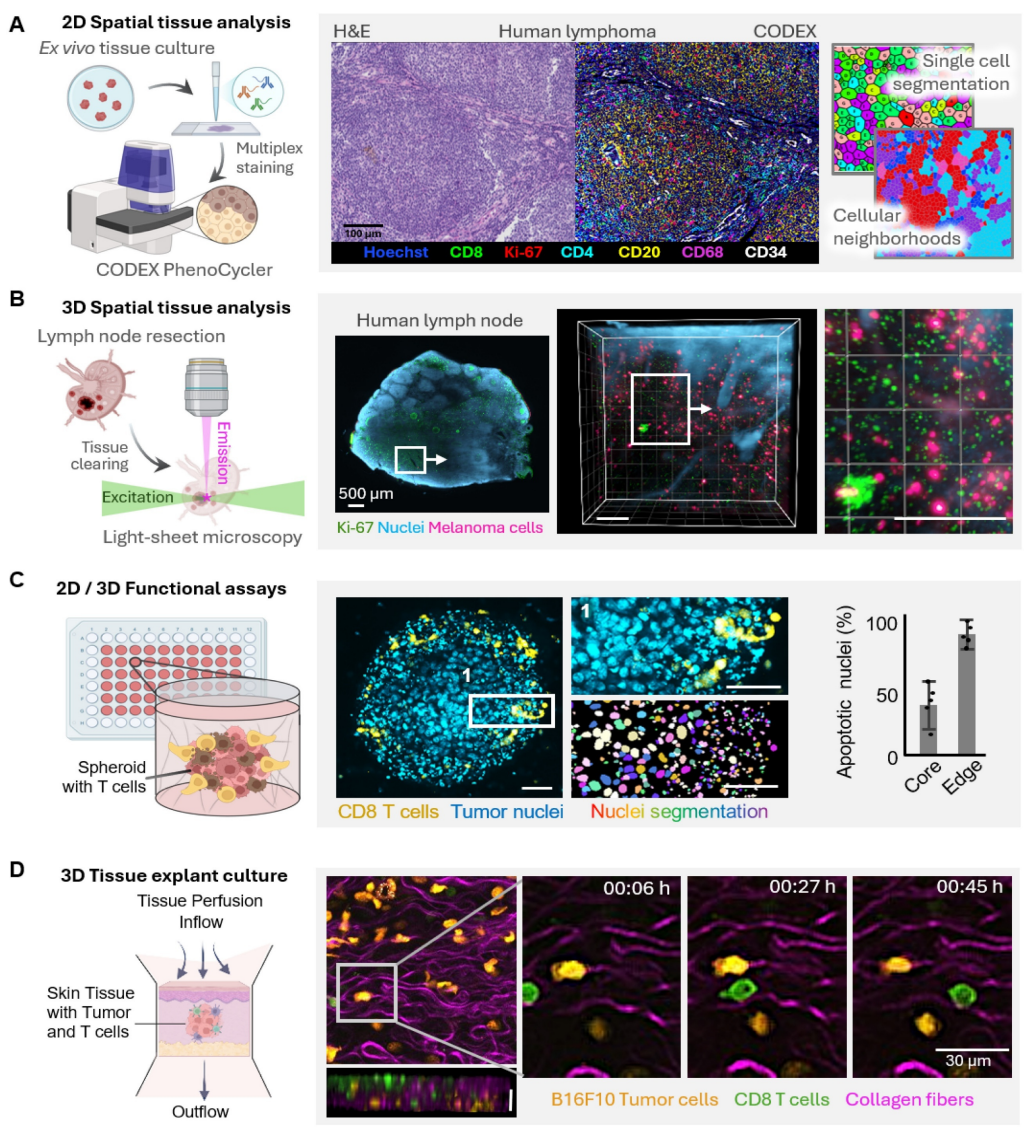
** Evolving analytical strategies for spatial and functional characterization of complex tumor models.** As cancer models advance from simple monolayers to complex multicellular systems, analytical tools must equally evolve to capture the increasing spatial and functional. **(A)** 2D spatial tissue analysis enables high-dimensional mapping of immune cell phenotypes within complex tumor models. Representative images illustrate immune infiltration patterns of immune cells in human lymphoma, with single-cell segmentation revealing phenotypic diversity and cellular neighborhoods. Scale bar, 100 µm. **(B)** Tissue clearing and light-sheet microscopy enables 3D spatial analysis and resolves the volumetric organization of tumor models and associated immune structures at cellular resolution. Shown is a resected and cleared human lymph node imaged by light-sheet microscopy, revealing infiltration with melanoma cells. Scale bars, 200 µm. **(C)** Dynamic functional assays based on live imaging of 3D tumor spheroids provide functional readouts in real-time. Here, a B16F10 melanoma spheroid is infiltrated by CD8⁺ T cells (green). Quantitative analysis of apoptotic nuclei (cyan) shows that killing efficacy is highest at the spheroid periphery, whereas tumor cells in the hypoxic core resist immune attack. Scale bars, 100 µm. **(D)** *Ex vivo* tissue explant cultures preserve the structural integrity and TME of intact tissue while enabling time-resolved observation of immune-tumor cell interactions. Multiphoton imaging of mouse skin explants containing melanoma cells (magenta) and CD8⁺ T cells (green) illustrates cytotoxic T cell infiltration and tumor-immune cell interactions and a z projection depicting a cross section of the tissue. Scale bar, 30 µm.

Given that cancer models transitioning from 2D monolayers to complex multicellular 3D systems and *ex vivo* tissues have developed, analytical methodologies must evolve in synchrony to accommodate their dynamic spatial and functional complexity **(Figure 3)**. High-content microscopy and multiplex immunofluorescence currently provide high-dimensional mapping of immune cell phenotypes and spatial relationships in tumor models, elucidating infiltration patterns and cellular neighbourhoods **(Figure 3A)**. In intact tissues, tissue clearing together with light-sheet microscopy enables the volumetric visualization of the tumor and immune architectures at cellular resolution **(Figure 3B)**. Dynamic live-cell imaging of 3D spheroids allows time-resolved readouts of immune-tumor interactions showing, among other things, spatial gradients in cytotoxic efficacy between spheroid peripheries and hypoxic cores **(Figure 3C)**. Multiphoton imaging of *ex vivo* tissue explants, in addition, allows for the preservation of native TMEs, provides access to the migration, infiltration, and tumor-immune contact dynamics in real time, complementary to the TMEs methods described **(Figure 3D)**. Taken together, these emergent imaging techniques enable a holistic, spatiotemporal, and functional outlook of cancer models, linking molecular mechanisms to therapeutic effectiveness, and advance their status as quantitative theranostic platforms at the crossroads of experimental oncology and clinical translation.

These spatially resolved readouts provide dual value in theranostics by functioning as diagnostic tools, identifying predictive tissue signatures of response, and as mechanistic assays that guide therapeutic optimization. For instance, correlating PD-L1 expression patterns with T cell localization in treated tumor slices can clarify why certain regions resist immune attack. Integrating these spatial data with transcriptomic or metabolomic profiles enables multiscale models of treatment efficacy. Ultimately, applying these technologies to patient-derived explants transforms them into quantitative theranostic platforms, thereby linking drug exposure, biological mechanism, and clinical outcome in one experimental system.

## From modeling to decision-making

Across a spectrum of cancer models from rapid 2D assays to complex *in vivo* xenografts, each platform addresses a different stage of the translational continuum. Simple models offer speed and scalability for early drug triage, whereas complex 3D systems deliver patient-specific biology at high spatial and temporal resolution. Such a blend of strategies in the context of FPM provides both throughput and physiological relevance. For theranostic purposes, the model itself becomes part of the diagnostic process and works as a living sensor reporting functional responses to therapy in real time. The selection of the best model is in turn subject to answering the clinical question: Is a rapid cytotoxicity screen needed for immediate therapy selection, or is an immune-competent tissue assay required to predict the efficacy of checkpoint blockade? This comprehensive perspective paves the way for the next generation of *ex vivo* culture platforms that aspire to combine clinical practicality with microenvironmental fidelity.

The ultimate vision reflects the theranostic principle by linking diagnostics directly to therapeutic decision-making. Utilizing patient-derived 3D models as personalized avatars, they are able to predict therapeutic benefit, explain resistance mechanisms, and identify companion biomarkers in this paradigm. As bioengineering, imaging, and computational analytics become increasingly intertwined, these living tumor systems will further cement the FPM role in creating a bridge between preclinical modelling and bedside decision-making.

Across different platforms, these models also serve important roles in the development and evaluation of diagnostic tools and theranostic agents. 2D cell cultures support high-throughput screening that can be used for early diagnostic triaging, and rapid cytotoxicity readouts such as LDH-release assays can quickly identify drug sensitivity profiles that guide early treatment decisions 36. 3D spheroids and PDOs enable real-time imaging of drug distribution, retention, and treatment response, providing functional readouts that more closely approximate *in vivo* behavior 79. Scaffold-based models can recapitulate specialized tissue structures such as skin, bone, or other organ-specific architectures, offering platforms for validating structure-dependent scenarios and localized theranostic delivery strategies 51. Microfluidic systems further enable dynamic monitoring of drug perfusion, biomarker release, and cell-cell interactions under controlled flow conditions, making them valuable for testing pharmacokinetics-linked diagnostic markers and evaluating tracer transport 80. *Ex vivo* tissue piece cultures and xenograft models preserve the native TME, allowing assessment of immune-tumor interactions, immune-cell infiltration, and TME-dependent imaging signatures 81. Together, these complementary model systems create a translational continuum for optimizing diagnostic modalities and theranostic approaches, from rapid drug-sensitivity assessment to biologically faithful validation of imaging-based therapeutic monitoring.

**Figure 4:**
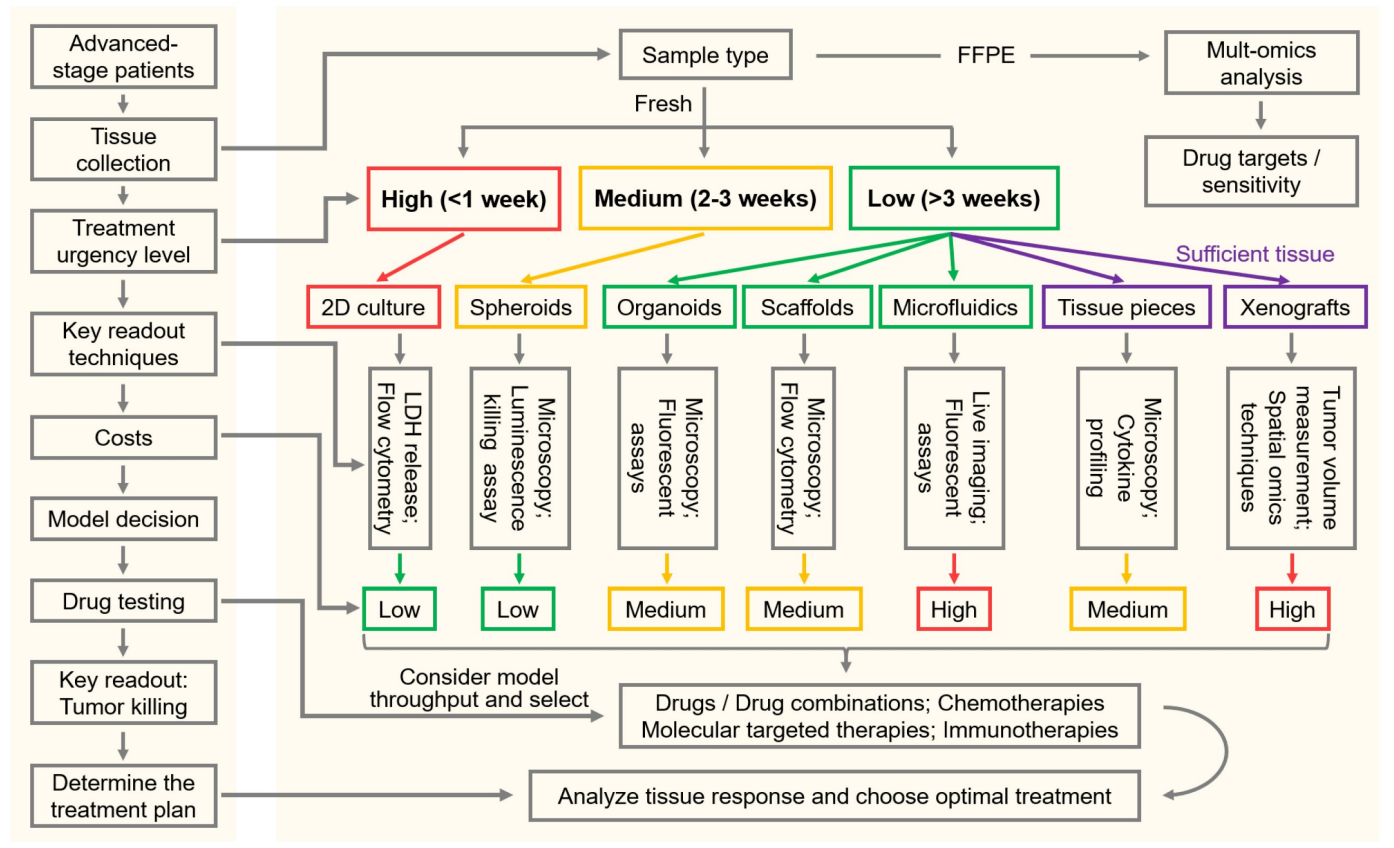
** Model-selection workflow for FPM.** Flowchart guides tumor model selection by linking clinical urgency, tissue availability, readouts and costs to support FPM decisions. Multiomics analysis of FFPE tissues enable the characterization of the immune TME to guide immunotherapy selection as well as the identification of drug or antibody targets on tumor cells. While antitumoral killing assays of fresh tissue will be used for treatment sensitivity predictions. Treatment urgency (high, medium and low) determines culture models. Sufficient tissue quantity enables testing with *ex vivo* tissue pieces culture or xenografts. Each model is paired with its suitable readout technologies and costs. Left: general workflow; right: detailed workflow.

To support practical decision-making in FPM, we provide a model-selection guiding flowchart that integrates clinical urgency, tissue availability, assay requirements, and model complexity (**Figure 4**). The workflow begins with the patient's clinical question, typically the need to identify an effective therapy within a defined treatment window, and links this directly to the type of tissue obtained (fresh vs. FFPE) and the amount available. For cases with very high treatment urgency (<1 week), rapid, low-cost 2D cultures, which can deliver fast cytotoxicity or flow cytometry-based readouts should be prioritized. For a window of 2-3 weeks, simple 3D systems like spheroids can be used, which maintain reasonable turnaround times and potentially improved readouts compared to 2D cultures. Low-urgency scenarios (>3 weeks) allow the use of more complex systems, such as organoids, scaffold-based models and microfluidic CoC platforms, in which live microscopy can be applied, e.g., to identify T-cell motility in response to immunotherapies. When larger tissue resections are available, *ex vivo* tissue pieces or xenografts can be performed, which provide the highest physiological relevance including intact TME architecture and tumor heterogeneity. At each step, the flowchart pairs available models with recommended readouts to assess tumor cell killing and antitumoral (immune) mechanisms, such as real-time microscopy, fluorescent assays, cytokine measurements, or tissue-level biomarkers. It also indicates cost and throughput to facilitate a balanced selection. By presenting these options side-by-side, the guide offers default choices for common clinical situations.

## Integration, translation and innovation

As cancer modelling continues to evolve, the next step is not only the creation of ever more complex systems, but the integration of existing models into coherent translational pipelines. Rapid 2D and spheroid assays can serve as front-line functional screens, while organoids, perfused explants, and xenografts provide higher-fidelity validation. The incorporation of immune components and advanced imaging makes these models particularly relevant for personalized immunotherapy. Standardization and scalability remain essential for the routine clinical application of these tools. Protocols for tissue handling, culture conditions, and data interpretation must be harmonized across centers.

To support immunotherapy evaluation across diverse experimental systems, **Table 2** provides a practical summary of how immune cells can be incorporated into common 2D, 3D,* ex vivo*, and *in vivo* tumor models. For each platform, we outline straightforward co-culture starting procedures, key time-course readouts, including infiltration, killing activity, and cytokine changes, and frequent technical challenges with troubleshooting solutions. Presenting these considerations side-by-side offers an accessible operational guide to help researchers select and implement the most suitable immune-competent model for functional precision immunotherapy studies.

In efforts to enhance reproducibility, we outline recommendations for feasible actions to decrease batch-to-batch variation across tumor model systems. For 2D cultures, ensuring strictly controlled seeding density and the use of aliquoted master stocks significantly enhances consistency 82. When producing spheroids, plate format, cell aggregation time, and initial cell counts should be standardized to minimize this variability by batch 83. Organoid variability may be reduced by establishing well-defined protocols for tumor tissue dissociation, ECM embedding, serial passaging, and morphological assessment, as well as applying strict quality control programs 84. The variability of scaffold and microfluidic models can be minimized by using commercially standardized fabrication parameters and calibrating flow rates before each experiment 85. For tissue slices, consistency improves when cuts are made using automated vibratomes at fixed thickness and when sampling is taken from equivalent tumor regions. While xenografts inherently demonstrate a greater biological variability, standardized implantation site, fragment size, or cell number and treatment schedules can increase the robustness of these assays 86. Incorporating these strategies enhances the reproducibility of studies and enables more accurate model selection.

In addition to traditional small molecule- and antibody approaches, new molecular platforms such as supramolecular systems and DNA-based nanotechnology have begun to gain significance in cancer theranostics. Supramolecular systems are organized structures formed through reversible, non-covalent molecular interactions, and can form dynamic assemblies with advantages including low immunotoxicity, target delivery and controlled release, making them well suited for precision medicine applications 87. Another example is DNA nanostructures, such as DNA origami, enable programmable spatial organization of therapeutic and diagnostic components, supporting precise delivery, targeting and imaging 88. When evaluated in FPM models, these platforms provide powerful tools for the systematic assessment of patient-specific uptake, efficacy, and toxicity, while the integration of advanced culture models with these technologies represents a promising direction for future innovation.

## Ethical and governance considerations

The use of patient-derived material in FPM also raises important ethical and governance issues. In most settings, participation requires written informed consent that specifies how fresh tissue will be used for *ex vivo* testing, what level of clinical interpretation can be expected, and under what conditions data may be shared. All assay-derived data should be stored on secure, access-controlled institutional servers and handled in accordance with local data protection regulations 97,98. Functional readout interpretation is typically carried out by a multidisciplinary tumor board or equivalent oversight group to ascertain that experimental findings are contextualized against known clinical, pathological, and genomic information. Due to the uncertainty intrinsic in functional assays, results should be communicated to clinicians and patients in unambiguous, standardized language, and avoid overstating predictive value 99. The implementation of these ethical and governance checks is a prerequisite to the responsible and clinically meaningful application of FPM approaches.

## Summary

FPM has shown substantial clinical utility, and the rapid advancement of culture technologies has greatly expanded its application potential. However, translation of complex cancer models into widespread FPM clinical practice is not without its challenges. Importantly, complexity of a model should not be sought simply for its own sake. Although advanced models like scaffold-based cancer models, tumor explants and PDXs present better representations of the biology of tumors, the complexity and cost in this approach render them less generally applicable to the clinic but still powerful tools in preclinical research. Simpler methods such as 2D suspension cultures, however, remain essential, particularly for the FPM application of hematological malignancies, for their cost-effectiveness and time value with predictive potential in clinical practice. In the case of solid tumors and immunotherapy, however, maintenance of the stromal and immune elements is key, as therapeutic success is often contingent upon the preservation of TME interactions. In this regard, 3D spheroids and organoids are the currently most established systems for performing FPM studies, and *in vivo* validation by PDXs complementarily supports this development. Recent advancements such as hybrid 3D constructs, and perfused tissue explant or bioreactor systems, continue this momentum by sustaining complex human tissues *ex vivo* for functional evaluation and mechanistic investigation.

In summary, FPM is advancing rapidly, and the growing array of cancer models brings many new possibilities while posing ongoing challenges for standardization and clinical integration. The next step will be harmonized standards for model selection and analytical readouts, ensuring that these innovations can be integrated into routine precision oncology and theranostic practice.

## Future outlook

As new anticancer agents continue to be added to the treatment pipeline, it is becoming ever more critical to determine which treatment is the best for each patient. Analogous to the development of antibiotic medications, antibiotics such as penicillin and other agents have existed and remain evidence-based treatment options; however, the increasing presence of a spectrum of antibiotics as well as antibiotic resistance has accelerated the implementation of antimicrobial susceptibility testing, leading to more targeted activity against susceptible agents 100. All indications now, excluding surgery, for treatment of cancer generally follow the same standard protocols followed for the treatment of the malignancy and there is typically a progression of cancer with first-line, second- and third-line drugs in the event that other cancer therapies fail. Although this progressive approach has historically helped the vast majority of patients, this stepwise pathway has led to some patients missing out on receiving suitable pharmacotherapies 101. FPM, which offers a rapid and direct method of matching drugs to patient-specific responses, holds great promise for transforming this paradigm and personalizing cancer treatment more effectively in the future. When integrated with imaging and spatially resolved analyses, FPM could evolve into a fully theranostic approach by linking predictive diagnostics directly to therapeutic decision making.

FPM has demonstrated great adaptability and strong benefits in hematologic malignancies owing to the relative ease of sampling and model development. Its remarkable improvement of treatment accuracy and effectiveness is already documented in several prospective studies and can help initiate a wider range for therapeutic incorporation 22,36. The question is how to maximize this success into immunotherapy of solid tumors where TME complexity and analytical depth are required.

Moving forward, it will be essential to establish standardized protocols and readouts, which are critical for integrating FPM into routine clinical practice across cancer types. Applying FPM to solid tumors remains particularly challenging. Very few prospective studies have been conducted, and researchers must identify suitable models for different tumor types that are both practical and cost-effective 102. Nonetheless, growing evidence from preclinical and co-clinical studies suggests that FPM in solid tumors is steadily advancing toward clinical translation. Future efforts should integrate immune-competent 3D and *ex vivo* tissue platforms with quantitative spatial and functional readouts, ensuring that the next generation of FPM assays not only predict response but also explain it, thereby realizing the vision of cancer theranostics.

## Figures and Tables

**Table 1:** Clinical and Preclinical Use of Patient-Derived Cancer Models in FPM

Cancer	Patient Samples	Study Type	Model & Approach	Major Significance	Ref.
AML	133/78/41 diagnosed/ relapsed/refractory	Prospective, clinical trial	2D-Suspension, 515 drugs screened	Led to 59% response rate in R/R AML, including 45% complete remission	Malani *et al.* 36
Hematologic Cancers	143 patients, and 56 treated based on FPM	Prospective, clinical trial	2D-Suspension, Single-cell FPM, 139 drugs screened	54% of patients showed improved PFS, with 40% experiencing prolonged responses	Kornauth *et al.* 22
AML	28 patient samples, FPM study	Prospective, clinical trial	2D-Suspension, 187 drugs screened	Induces clinical responses in progressive AML and predicts emerging drug resistance	Pemovska *et al.* 35
Solid Tumors	568 biopsies of various cancer types	Prospective, co-clinical trial	2D-Monolayer, 6 TKIs tested	Quantify drug sensitivity and reflect clinical response	Kodack *et al.* 38
Pediatric Cancers	21 R/R patients, FPM study	Prospective, clinical trial	2D cell cultures, 125 drugs screened	5 cases out of 6 experienced an improvement in PFS, after FPM-guided treatments	Acanda *et al.* 10
Lung Cancer	20 biopsies of lung cancer	Prospective, co-clinical trial	3D-Spheroids, 6 TKIs tested	Strong predictive performance in co-clinical trials, with an accuracy of 85%	Shie *et al.* 42
Ovarian Cancer	44 newly diagnosed eligible patients	Prospective, co-clinical trial	3D-Spheroids, 6 chemotherapy drugs	High overall prediction of clinical response accuracy of 89%	Shuford*et al.* 43
mGI cancers	29 biopsy samples from 21 patients	Prospective, co-clinical trial	3D-Organoids, 55 drugs screened	High predictive accuracy, 100% sensitivity and 93% specificity	Vlachogiannis *et al.* 48
Breast cancer	35 patients	Prospective, co-clinical trial	3D-Organoids, 49 drugs screened	Predicting clinical response with 82.35% sensitivity and 69.23% specificity	Chen *et al.* 49
Breast cancer	2 patient samples	Pre-clinical study	Polymer Scaffold, 2 chemotherapy drugs	Discover heterogeneity in drug response	Nayak *et al.* 54
Prostate cancer	2 patient samples	Pre-clinical study	3D-Hydrogels, Docetaxel treated	Demonstrated potential for drug screening	Fong *et al.* 55
CRC	23 patient samples	Pre-clinical study	Tissue scaffolds, 5-FU and ​FOLFIRI	Serve as a patient-specific platform for drug testing	Sensi *et al.* 56
Breast cancer	2 patient samples	Pre-clinical study	Cancer-on-a-chip, CAR T cell therapy	Enables personalized CAR-T efficacy and safety evaluation	Maulana *et al.* 60
Lung Cancer	12 patient samples	Pre-clinical study	Cancer-on-a-chip, Anti-PD-1 therapy	Enables assessment of patient-specific anti-PD-1 response	Veith *et al.* 61
GBM	7 grade IV samples	Pre-clinical study	Tumor fragments, 2 Immunotherapies	Enables multidimensional personalized assessment of immunotherapy response	Shekarian *et al.* 62
PDAC	3 patient samples	Pre-clinical study	Tumor Slices Chemotherapy	Enables chemotherapy testing and personalized therapy assessment	Hughes *et al.* 65
Gallbladder cancer	12 patients treated based on FPM	Prospective, clinical trial	Xenografts, 5 chemotherapy drugs	PDX-guided chemotherapy significantly upregulated OS and DFS of patients	Zhan *et al.* 72
HNSCC	49 patient samples, PDX clinical trial	Pre-clinical study	Xenografts, Cetuximab tested	Serve as a patient-specific platform for testing drug sensitivity	Yao *et al.* 73

**Abbreviations: AML**, Acute Myeloid Leukemia; **R/R**, Relapsed/Refractory; **PFS**, Progression-Free Survival; **TKIs**, Tyrosine Kinase Inhibitors; **mGI** cancer, metastatic gastrointestinal cancer; **CRC**, Colorectal Cancer; **GBM**, Glioblastoma Multiforme; **PDAC**, Pancreatic Ductal Adenocarcinoma; **OS**, Overall Survival; **DFS**, Disease-Free Survival; **HNSCC**, Head and Neck Squamous Cell Carcinoma.

**Table 2:** Immune co-culture workflow, readouts, and troubleshooting across cancer models

Model	Basic Co-culture Setup	Key Readouts	Common Issues	Troubleshooting
2D Cell Culture	Tumor cells in plate and add activated T cells (Chakraborty *et al.* 89)	Killing assays such as LDH release and flow cytometry, cytokine assay by ELISA	Over-rapid cancer cell killing, non-physiological interactions, not ideal for immunotherapy test	Lower E:T ratio, reduce activation strength or only for non-immunotherapies
3D Spheroids	Form spheroids with ultra-low attachment plates or hydrogel matrix and co-incubate immune cells (Lo *et al.* 90)	Live-cell imaging of infiltration, confocal microscopy, apoptosis markers, viability dyes	Limited immune infiltration, spheroid size variability	Standardize spheroid size, optimize matrix stiffness
Organoids (PDOs)	Grow tumor cells and peripheral T cells or NK cells in Matrigel domes (Sun *et al.* 91)	Imaging-based killing assays, cytokines, single-cell profiling, flow cytometry after dissociation	Dense Matrigel barrier, contamination by fibroblasts, slow establishment	Use diluted ECM, mechanical dissociation for uniformity, pre-expand TILs
Scaffold-based Models	Seed tumor cells in scaffolds and add immune cells (Mistretta *et al.* 92)	Infiltration depth, matrix remodeling, live imaging, killing assays	Immune cells trapped at scaffold surface; irregular perfusion	Adjust pore size, reduce hydrogel density, use dynamic flow
Microfluidic CoC	Load tumor cells and other TME components into microfluidic channels (Ao *et al.* 93)	High-resolution monitoring of infiltration dynamics, killing, cytokine gradients	Channel clogging; shear-stress-induced immune dysfunction	Reduce cell density, optimize flow rate, pre-coat channels with ECM
Tissue Pieces (*Ex vivo* Slices)	Culture 250 µm tumor slices (Jiang *et al.* 94) or tissue pieces (Zhang *et al.* 66) to maintain TME for immunotherapy assays	Multiphoton imaging (motility, contacts), histology, multiplex microscopy, cytokine secretion	Rapid tissue decay; poor penetration of immune cells	Use oxygenated media, maintain <48h, cut thinner slices, embed in agarose
Xenografts / PDX	Engraft tumor (immunodeficient mice for adoptive transfer of T cells, Stenger *et al.* 95) or humanized mice (Meraz *et al.* 96)	*In vivo* immune cell infiltration (histology, flow cytometry), tumor size, serum cytokines	Human T-cell exhaustion; variability between animals	Use fresh T cells, optimize dosing schedule, include ≥5-6 mice per group

ECM, extracellular matrix; ELISA, enzyme-linked immunosorbent assay; E:T, effector:target; LDH, lactate dehydrogenase; NK cells, natural killer cells; TILs, tumor-infiltrating lymphocytes; TME, tumor microenvironment; PDOs, patient-derived organoids; PDX; patient-derived xenografts

## Abbreviations

2D: two-dimensional
3D: three-dimensional
TME: tumor microenvironment
ECM: extracellular matrix
PDX: patient-derived xenografts
FPM: Functional Precision Medicine
PDOs: patient-derived organoids
LDH: lactate dehydrogenase
AI: artificial intelligence
AML: acute myeloid leukemia
LLPS: liquid-liquid phase separation
5-FU: 5-fluorouracil
FOLFIRI: folinic acid, fluorouracil, and irinotecan
CoC: Cancer-on-a-Chip
